# Secondary Hemophagocytic Syndrome Associated with Richter's Transformation in Chronic Lymphocytic Leukemia

**DOI:** 10.1155/2014/287479

**Published:** 2014-01-16

**Authors:** Nura El-Haj, Wilson I. Gonsalves, Vinay Gupta, Jacob P. Smeltzer, Sameer A. Parikh, Preet P. Singh, Naseema Gangat

**Affiliations:** ^1^Trinity College, Dublin, Ireland; ^2^Department of Hematology, Mayo Clinic Rochester, 200 1st Street SW, Rochester, MN 55905, USA

## Abstract

Hemophagocytic syndrome (HPS) is an extremely rare condition arising from the overactivation of one's own immune system. It results in excessive inflammation and tissue destruction. Prompt initiation of treatment is warranted in either scenario in order to decrease mortality. Most cases are triggered by infectious agents, malignancy, or drugs. We describe the first case of a CLL patient presenting with HPS due to acquisition of EBV-related large cell lymphoma in the setting of profound immunodeficiency.

## 1. Background

Hemophagocytic syndrome (HPS) is an extremely rare but potentially fatal hyperinflammatory condition arising from the overactivation of one's own immune system. It can be classified as either primary or secondary [1, 2]. Primary HPS, also known as familial hemophagocytic lymphohistiocytosis (FHL), is an autosomal recessive disorder caused by the inheritance of gene mutations producing defective regulatory proteins responsible for the cytolysis of abnormal immune cells [2, 3]. Secondary HPS is caused by strong self-immunologic activation that is mostly not hereditary but arises due to triggers in the setting of conditions such as autoimmune diseases, immunodeficiency, underlying malignancy or disseminated infections from viruses, fungi, or bacteria [2, 4, 5]. However, approximately 15% of secondary HPS patients have an underlying hereditary predisposition to develop HPS. Either form of HPS results in excessive inflammation and tissue destruction. Prompt initiation of treatment is warranted in either scenario in order to decrease mortality [1].

On the other hand, chronic lymphocytic leukemia (CLL) is the most common leukemia in the Western world with a heterogeneous and mostly prolonged disease course [6, 7]. CLL patients are immunodeficient to a degree, with a significant proportion being hypogammaglobulinemic [8, 9]. Furthermore, profound defects of cell-mediated immunity can more readily occur in those treated with purine analogues [10]. Secondary HPS has been sparsely reported in association with CLL. However, most cases were triggered by an infective agent, such as EBV, histoplasmosis, or H1N1 influenza [5, 11–14], by the chemotherapeutic drugs used in the treatment of CLL such as fludarabine or rituximab [4] or by the direct progression of CLL itself [15, 16]. To date there have been no cases in the literature reporting a CLL patient presenting with a secondary HPS due to acquisition of EBV related large cell lymphoma in the setting of profound immunodeficiency as described in this case report.

## 2. Case Presentation 

A 57-year-old Caucasian male was diagnosed with asymptomatic Rai stage I CLL during the pathological evaluation of his pelvic lymph nodes following a radical prostatectomy performed for the treatment of his prostate cancer. His CLL remained asymptomatic for several months, but eventually required systemic chemoimmunotherapy with rituximab and fludarabine due to progressive abdominal and mediastinal lymphadenopathy. Over the course of the following seven years, progression of CLL-associated lymphadenopathy warranted sequential treatment with several regimens that included alemtuzumab, lenalidomide, chlorambucil, and lastly, a combination of rituximab with bendamustine, all of which provided mostly short-term responses.

Despite mild radiographic responses of his CLL related lymphadenopathy to his last dose of the chemoimmunotherapy combination of rituximab and bendamustine, he continued to suffer from progressive lethargy, decreased appetite, episodes of intermittent altered mental status, and new onset fevers of unknown origin for two consecutive months. He was evaluated extensively at an outside health care facility for an infectious cause of his symptoms. Although no cause was detected, he was found to be hypogammaglobulinemic and was subsequently treated with monthly intravenous gammaglobulin therapy.

He presented to our facility for a second opinion and further assessment of his persistent symptoms. In view of progressive dyspnea, fevers, and altered mental status, he was directed to the emergency department (ED) prior to any outpatient evaluation. On subsequent evaluation in the ED, he was found to be hypotensive (blood pressure: 89/65), hypoxic (O2 saturation at room air: 85%), and hyponatremic (sodium: 126) in the setting of an acute kidney injury. He was admitted to the intensive care unit and was initiated on broad-spectrum antibiotics that included vancomycin, cefepime, and metronidazole, while being aggressively hydrated with intravenous fluids to correct his hypotension and hyponatremia.

**Figure 1:**
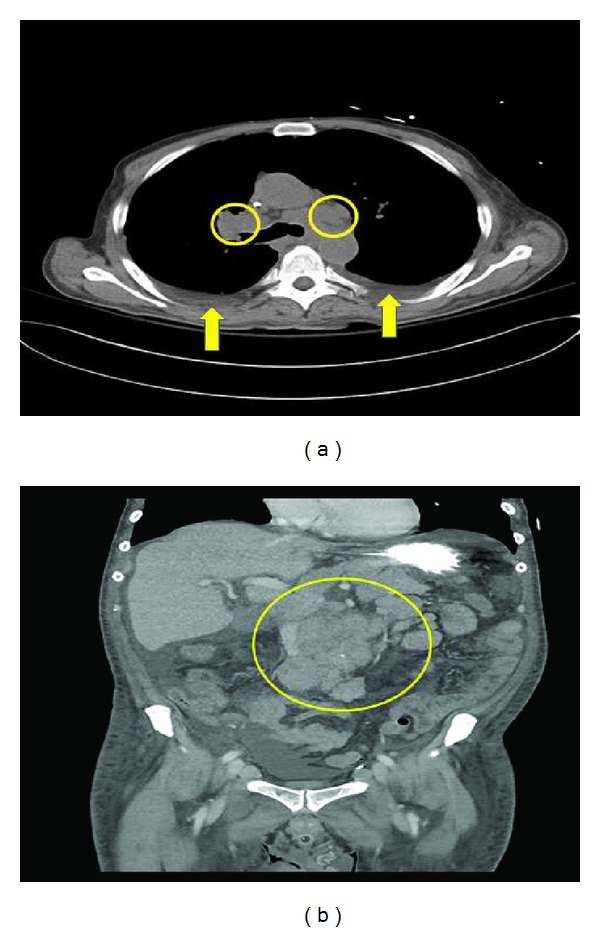
(a) CT imaging of the chest demonstrating bilateral pleural effusions (arrows) and mediastinal adenopathy (circles) and (b) CT imaging of the abdomen demonstrating bulky mesenteric adenopathy (circle).

**Figure 2:**
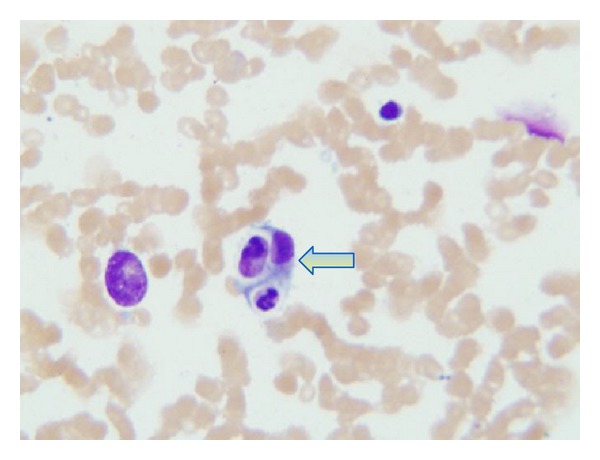
Bone marrow aspirate demonstrating a blue foamy macrophage engulfing erythroid and lymphoid precursors (arrow).

**Figure 3:**
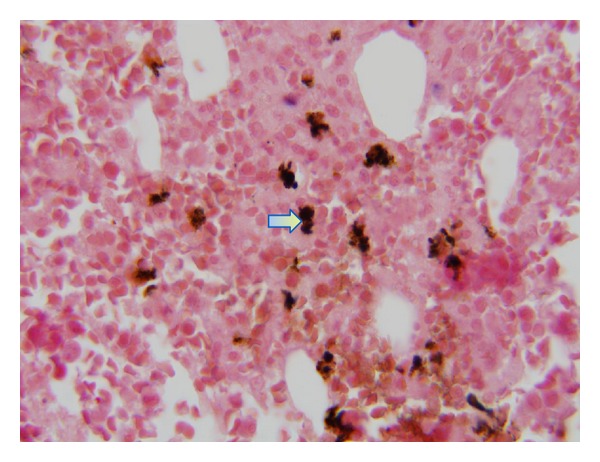
Bone marrow biopsy demonstrating a cluster of large cells positive for the EBV-encoded RNA (EBER) stain suggestive of an EBV driven large cell lymphoma (arrow).

Computed tomography imaging of his chest and abdomen revealed significant thoracic and upper abdominal lymphadenopathy, bilateral pulmonary nodules, and pleural effusions (Figures 1(a) and 1(b)). An extensive workup to rule out fungal infections was negative for histoplasmosis, pneumocystis, *Aspergillus*, blastomycoses, coccidiomycosis, and *Cryptococcus*. In addition, viral PCR for HIV, hepatitis A, B, and C, VZV, adenovirus, influenza, and RSV was negative except for EBV with the PCR detecting 15,900 copies in the peripheral blood. During this time, the patient decompensated further requiring ventilatory, hemodialysis, and pressor support. He became progressively pancytopenic with a white blood count of 1.9 × 10^9^/L (3.5–10 × 10^9^/L), hemoglobin of 10.0 g/dL (normal range 13.5–17.5 g/dL), and platelets of 29 × 10^9^/L (150–450 × 10^9^/L). Given the acute pancytopenia and clinical decompensation, a bone marrow biopsy and aspirate were performed and showed a moderately hypocellular marrow of 25% with large amounts of diffuse histiocytes demonstrating hemophagocytosis and marked decrease in erythroid, megakaryocytic, and granular precursors (Figure 2). Moreover, there was an abnormal population of CD5+ and CD23+ small monoclonal B-cells congruous with CLL as well as a population of large neoplastic cells that were CD20+, CD79a+, PAX-5+, CD5−, CD23−, CD30+, CD15−, CD45−, and EBV-encoded RNA (EBER) positive (Figure 3). These findings were consistent with an EBV+ large B cell lymphoma. Further laboratory investigations revealed the following levels: ferritin 8780 mcg/L (24–336 mcg/L), fibrinogen 440 mg/dL (200–375 mg/dL), and triglycerides 262 mg/dL (<150 mg/dL); NK cytotoxicity at effector: target cell ratio 100 : 1 = 60% and 25 : 1 = 31% (normal range for 84 healthy donors, average % cytotoxicity at E : T ratio, 100 : 1 = 44%; 25 : 1 = 20%) and sIL-2r 30,567 units/mL (45–1,105 units/mL). These results together with his age and lack of previous similar symptoms suggested a secondary HPS. Considering that the EBV PCR blood titers were only 15,900 copies/mL, which is not a level usually associated with an EBV driven hemophagocytic process, it was believed that this patient most likely acquired an EBV driven large cell lymphoma as a consequence of being immunocompromised from his CLL and having received multiple chemotherapy treatments; thus, we believe that his lymphoma was possibly triggering this HPS. Antimicrobial therapy was stopped in the setting of persistently negative blood cultures and the patient was given corticosteroids of 1 gram of methylprednisolone to dampen down the brisk immune response. Unfortunately, the patient was in no physical condition to withstand further chemoimmunotherapy. He quickly developed bacteremia and septic shock from gram-positive cocci resembling streptococci. Given the grave prognosis and in accordance with his family's wishes, comfort care measures were undertaken and he passed away shortly thereafter.

## 3. Discussion 

HPS is thought to involve erratic behavior of the immune system by its inability to downregulate an immune response or failure to clear antigens, resulting in persistent stimulation of macrophages and cytotoxic cells. Subsequent cytokinemia drives the proliferation of the monocyte-macrophage system causing organ infiltration, which eventually causes multiorgan failure [5]. Elevated levels of a marker of T cell activity, soluble IL-2 receptor (sIL2r or sCD25), have been shown to correlate with prognosis; prompt diagnosis and initiation of therapy are necessary to prevent irreversible tissue damage [2, 15].

The criteria enumerated by the International Histiocyte Society for the diagnosis of HPS include (1) fever, (2) splenomegaly, (3) cytopenias affecting at least two of three lineages in peripheral blood, (4) hypertriglyceridemia and/or hypofibrinogenemia, and (5) hemophagocytosis in the bone marrow, spleen, or lymph nodes. Since the elaboration of these diagnostic criteria, additional criteria have been introduced: (6) low or absent NK-cell activity, (7) hyperferritinemia, and (8) high levels of sIL-2r [17]. Evidence of immunopathology has been reported to include liver dysfunction with portal triaditis and consumptive coagulopathies, CNS dysfunction, and progression to respiratory and kidney failure [2]. Although the presence of multiple criteria reflects the severity of the condition, some patients may not initially present with those criteria. Thus the entire clinical picture needs to be taken into account to formulate a diagnosis [2, 3].

In this case report, we describe a case of secondary HPS triggered by an EBV driven lymphoma in the context of CLL immunodeficiency. Mechanisms of CLL immunocompromise implicate humoral and cellular responses along with exacerbations related to immunosuppressive therapy. Tumoral cells act through cell-cell contacts and cytokine secretion to provoke an excessive T cell suppression, abnormal response to interleukin-2, and downregulation of immunoglobulin secretion by NK cells, which culminates in a failure in B lymphocytes regulation and decreased antibody production [8, 18, 19]. An alternative hypothesis explaining CLL immunodeficiency proposes that hypogammaglobulinemia results from the accumulation of neoplastic B cells that eventually dilute the number of normal immunoglobulin secreting B cells [20]. Hypogammaglobulinemia has been reported in up to 85% of CLL patients and subsequent infections are estimated to contribute to up to 50% of all CLL related deaths [8, 9, 18].

Following EBV infection, the virus can remain latent within host B cells primarily contained by cytotoxic T cells and NK cells [21, 22]. When the immune system fails to control EBV, infected carrier B cells can transform into malignant cells [21, 23]. EBV positive cells are sustained by programs of viral gene expression. Specifically, EBV latent membrane proteins adopt signaling pathways such as JAK/STAT and NF-*Κ*B, allowing B cell activation, differentiation, and survival [22, 24]. B cell immortalization contributes to development of carcinomas and lymphomas, as illustrated in this case report.

HPS has been reported in the literature to be associated with CLL [5, 11–16]. This case is unique in that we present an illustration of the possible relationship between acquired immunodeficiency, malignant transformation, and the development of a hyperinflammatory state, thereby highlighting the challenge in identifying the etiology of secondary HPS. In this case, HPS was not solely triggered by EBV, but rather by superimposition of infectious and malignant phenomena where CLL acquired immunodeficiency allowed for EBV to bring about Richter's transformation to a large cell lymphoma that manifested as HPS.

## References

[1] Janka G (2009). Hemophagocytic lymphohistiocytosis: when the immune system runs amok. *Klinische Padiatrie*.

[2] Weitzman S (2011). Approach to hemophagocytic syndromes. *Hematology*.

[3] Gupta A, Weitzman S, Abdelhaleem M (2008). The role of hemophagocytosis in bone marrow aspirates in the diagnosis of hemophagocytic lymphohistiocytosis. *Pediatric Blood and Cancer*.

[4] Tiong IS, Lau MBY, Toumoua S, Chiruka S (2012). A case of hemophagocytic lymphohistiocytosis in a patient with chronic lymphocytic leukemia after treatment with fludarabine, cyclophosphamide, and rituximab chemotherapy, with autopsy findings. *Case Reports in Hematology*.

[5] Rao RD, Morice WG, Phyliky RL (2002). Hemophagocytosis in a patient with chronic lymphocytic leukemia and histoplasmosis. *Mayo Clinic Proceedings*.

[6] Riches JC, Gribben JG (2013). Understanding the immunodeficiency in chronic lymphocytic leukemia: potential clinical implications. *Hematology/Oncology Clinics of North America*.

[7] Parker TL, Strout MP (2011). Chronic lymphocytic leukemia: prognostic factors and impact on treatment. *Discovery Medicine*.

[8] Freeman JA, Crassini KR, Best OG (2013). Immunoglobulin G subclass deficiency and infection risk in 150 patients with chronic lymphocytic leukemia. *Leukemia and Lymphoma*.

[9] Hamblin AD, Hamblin TJ (2008). The immunodeficiency of chronic lymphocytic leukaemia. *British Medical Bulletin*.

[10] Wadhwa PD, Morrison VA (2006). Infectious complications of chronic lymphocytic leukemia. *Seminars in Oncology*.

[11] Lai S, Merritt BY, Chen L, Zhou X, Green LK (2012). Hemophagocytic lymphohistiocytosis associated with influenza A, (H1N1) infection in a patient with chronic lymphocytic leukemia: an autopsy case report and review of the literature. *Annals of Diagnostic Pathology*.

[12] Chaker L, Segeren CM, Bot FJ, Maartense E (2010). Haemophagocytic syndrome and Hodgkin’s disease variant of Richter’s syndrome after fludarabine for CLL. *European Journal of Haematology*.

[13] van Koeveringe MP, Brouwer RE (2010). Histoplasma capsulatum reactivation with haemophagocytic syndrome in a patient with chronic lymphocytic leukaemia. *Netherlands Journal of Medicine*.

[14] Manoharan A, Catovsky D, Lampert IA (1981). Histiocytic medullary reticulosis complicating chronic lymphocytic leukaemia: malignant or reactive?. *Scandinavian Journal of Haematology*.

[15] Meki A, O'Connor D, Roberts C, Murray J (2011). Hemophagocytic lymphohistiocytosis in chronic lymphocytic leukemia. *Journal of Clinical Oncology*.

[16] Ando K, Miyazawa K, Kuriyama Y, Kimura Y, Mukai K, Ohyashiki K (2004). Hemophagocytic syndrome associated with CD8 positive T-cell chronic lymphocytic leukemia. *Leukemia and Lymphoma*.

[17] Henter J-I, Horne A, Aricó M (2007). HLH-2004: diagnostic and therapeutic guidelines for hemophagocytic lymphohistiocytosis. *Pediatric Blood and Cancer*.

[18] Nosari A (2012). Infectious complications in chronic lymphocytic leukemia. *Mediterranean Journal of Hematology and Infectious Diseases*.

[19] Tsiodras S, Samonis G, Keating MJ, Kontoyiannis DP (2000). Infection and immunity in chronic lymphocytic leukemia. *Mayo Clinic Proceedings*.

[20] Sampalo A, Brieva JA (2002). Humoral immunodeficiency in chronic lymphocytic leukemia: role of CD95/CD95L in tumoral damage and escape. *Leukemia and Lymphoma*.

[21] Roschewski M, Wilson WH (2012). EBV-associated lymphomas in adults. *Best Practice and Research*.

[22] Chen MR (2011). Epstein-barr virus, the immune system, and associated diseases. *Frontiers in Microbiology*.

[23] Grywalska E, Markowicz J, Grabarczyk P, Pasiarski M, Roliński J (2013). Epstein-Barr virus-associated lymphoproliferative disorders. *Postępy Higieny i Medycyny Doświadczalnej*.

[24] Pattle SB, Farrell PJ (2006). The role of Epstein-Barr virus in cancer. *Expert Opinion on Biological Therapy*.

